# Confirmatory factorial analysis of the Maslach Burnout Inventory – Human Services Survey in health professionals in emergency services*

**DOI:** 10.1590/1518-8345.3320.3386

**Published:** 2021-01-08

**Authors:** Sandra de Souza Pereira, Joana Fornés-Vives, Sara Guadalupe Unda-Rojas, Gerson Alves Pereira-Junior, Mario Francisco Juruena, Lucilene Cardoso

**Affiliations:** 1 Universidade do Estado de Minas Gerais, Unidade Passos, Passos, MG, Brazil.; 2 Universitat de les lles Balears, Departamento de Enfermería y Fisioterapia, Palma de Mallorca, Illes Balears, Spain.; 3 Universidad Nacional Autónoma de México, Facultad de Estudios Superiores Zaragoza, Ciudad de México, CDMX, Mexico.; 4 Universidade de São Paulo, Faculdade de Medicina de Ribeirão Preto, Ribeirão Preto, SP, Brazil.; 5 King’s College London, Department of Psychological Medicine, London, ENG, United Kingdom.; 6 Universidade de São Paulo, Escola de Enfermagem de Ribeirão Preto, PAHO/WHO Collaborating Centre for Nursing Research Development, Ribeirão Preto, SP, Brazil.

**Keywords:** Factor Analysis, Statistical, Psychometrics, Burnout, Psychological, Health Personnel, Emergencies, Stress, Psychological, Análise Fatorial, Psicometria, Esgotamento Psicológico, Pessoal de Saúde, Emergências, Estresse Psicológico., Análisis Factorial, Psicometría, Agotamiento Psicológico, Personal de Salud, Urgencias Médicas, Estrés Psicológico

## Abstract

**Objective:**

to confirm the factorial validity of the Maslach Burnout Inventory – Human Services Survey version in a sample of health professionals from the emergency services.

**Method:**

a quantitative, exploratory, descriptive and analytical study. Two hundred and eighty-two health professionals participated in the study. For data collection, a sociodemographic questionnaire and the Maslach Burnout Inventory were used. The psychometric sensitivity for the MBI-HSS items was estimated by measures of central tendency, variability and the distribution shape. Internal consistency was estimated using Cronbach’s alpha coefficient and the adequacy of the sample was verified using the Kaiser-Meyer-Olkin (KMO) index. As indexes for assessing the quality of fit of the model, the chi-square ratio by the degrees of freedom (χ^2^/DoF), the comparative fit index (CFI), the goodness of fit index (GFI), the Tucker Lewis index (TLI) and the root mean square error of approximation (RMSEA) were considered. To test data fit, the maximum likelihood method was used.

**Results:**

the three-factor structure of the Maslach Burnout Inventory was confirmed. Items 9, 12, 15 and 16 had a factorial weight below what was considered appropriate and were removed from the model. The second order hierarchical model with the aforementioned modifications presented an adequate adjustment to the data and can be considered the best and most parsimonious model tested according to the information theory indexes. The internal consistency of the instrument’s factors was recalculated considering the exclusion of the items and the three factors were considered adequate.

**Conclusion:**

the results obtained show that the Maslach Burnout Inventory is a reliable and factorially valid instrument for measuring the burnout syndrome in emergency service professionals in Brazil.

## Abstract (pt)

**Objetivo:**

confirmar a validade fatorial do *Maslach Bunout Inventory* – versão *Human Services Survey* numa amostra de profissionais da saúde dos serviços de emergência.

**Método:**

estudo de abordagem quantitativa, exploratório, descritivo e analítico. Participaram do estudo 282 profissionais de saúde. Utilizou-se para a coleta de dados, um questionário sociodemográfico e o *Maslach Burnout Inventory*. A sensibilidade psicométrica dos itens do *Maslach Bunout Inventory* – versão *Human Services Survey* foi estimada pelas medidas de tendência central, variabilidade e forma da distribuição. A consistência interna foi estimada por meio do Coeficiente alfa de Cronbach e a adequação da amostra verificada por meio do índice de Kaiser-Meyer-Olkin (KMO). Como índices para a avaliação da qualidade de ajustamento do modelo foram considerados a razão de qui-quadrado pelos graus de liberdade (χ^2^ /gl), *comparative fit index* (CFI), *goodness of fit index* (GFI), *índice de Tucker Lewis* (TLI) e *root mean square error of aproximation* (RMSEA). Para testar o ajustamento dos dados utilizou-se o método de máxima verossimilhança.

**Resultados:**

confirmou-se a estrutura trifatorial do *Maslach Burnout Inventory*. Os itens 9, 12, 15 e 16 apresentaram peso fatorial abaixo do considerado adequado e foram removidos do modelo. O modelo hierárquico de segunda ordem, com as modificações mencionadas, apresentou ajustamento adequado aos dados e pôde ser considerado o melhor e mais parcimonioso modelo testado de acordo com os índices da teoria da informação. A consistência interna dos fatores do instrumento foi recalculada considerando-se a exclusão dos itens e foi considerada adequada nos três os fatores.

**Conclusão:**

os resultados obtidos mostram que o *Maslach Burnout Inventory* é um instrumento confiável e fatorialmente válido para medir a síndrome de o *burnout* em profissionais dos serviços de emergência no Brasil.

## Abstract (es)

**Objetivo:**

confirmar la validez factorial del *Maslach Bunout Inventory* – versión *Human Services Survey* en una muestra de profesionales de la salud de los servicios de emergencia.

**Método:**

estudio cuantitativo, exploratorio, descriptivo y analítico. Participaron en el estudio 282 profesionales de la salud. Para la recopilación de datos, se utilizó un cuestionario sociodemográfico y el *Maslach Burnout Inventory* . La sensibilidad psicométrica de los ítems del *Maslach Bunout Inventory* – versión *Human Services Survey* se estimó mediante medidas de tendencia central, variabilidad y forma de distribución. La consistencia interna se estimó usando el Coeficiente alfa de Cronbach y la adecuación de la muestra se verificó usando el índice de Kaiser-Meyer-Olkin (KMO). Como índices para evaluar la calidad de ajuste del modelo fueron considerados la razón chi-cuadrado para los grados de libertad (χ ^2^ /gl), *comparative fit index* (CFI), *goodness of fit index* (GFI), índice de Tucker Lewis (TLI) y *root mean square error of aproximation* (RMSEA). Para probar el ajuste de los datos, se utilizó el método de máxima verosimilitud.

**Resultados:**

se confirmó la estructura de tres factores del *Maslach Burnout Inventory* . Los ítems 9, 12, 15 y 16 presentaron un peso factorial por debajo de lo que se consideraba adecuado y se eliminaron del modelo. El modelo jerárquico de segundo orden, con las modificaciones antes mencionadas, presentó un ajuste adecuado a los datos y puede considerarse el mejor y más parsimonioso modelo probado de acuerdo con los índices de teoría de la información. La consistencia interna de los factores del instrumento se recalculó ponderando la exclusión de los ítems y se consideró adecuada en los tres factores.

**Conclusión:**

los resultados obtenidos muestran que el *Maslach Burnout Inventory* es un instrumento confiable y factorialmente válido para medir el síndrome de *burnout* en los profesionales de los servicios de emergencia en Brasil.

## Introduction

The Maslach Burnout Inventory – Human Service Survey (MBI-HSS) stands out as the most used instrument to assess the burnout syndrome and its configuration in three dimensions has been confirmed, worldwide, in different populations. However, there is no Brazilian study that has investigated its validity in health teams from emergency services. Furthermore, it is relevant to develop analyses that prove that the items on a scale measure exactly what they propose.

This is a psychological syndrome that develops in response to chronic interpersonal stressors in the workplace^(^ 1 ^-^ 3 ^)^. It is characterized by emotional exhaustion (feelings of extreme tiredness related to excessive physical and emotional effort); depersonalization (negative attitudes in interpersonal relationships, marked by cynicism and disinterest) and low personal fulfillment (negative self-assessment of oneself, work ability, and to deal with other people)^(^ 1 ^,^ 4 ^-^ 7 ^)^.

The Maslach Burnout Inventory was developed in 1981, originally to be applied to human service professionals. The three dimensions that make up the scale emerged from exploratory items collected from interviews with health care professionals, with the aim of reflecting on the experiences related to the phenomenon^(^ 1 ^)^. With increasing interest in the burnout syndrome, other versions of this instrument have been developed^(^ 3 ^)^.

There are currently three versions of the Maslach Burnout Inventory: the Human Services Survey (MBI-HSS), used for the health services; the Educators Survey (MBI-ES), used in the educational area and the General Survey (MBI-GS), used for workers in general^(^ 8 ^)^. There are other assessment instruments, however, the MBI is the most used by the national and international scientific community, showing high reliability regardless of the sample^(^ 4 ^,^ 7 ^)^.

The MBI-HSS has 22 statements that comprise the frequency of feelings and attitudes towards clients and work. These statements are divided into three dimensions: *emotional exhaustion* (made up of nine items), *depersonalization* (made up of five items) and *personal fulfillment* (made up of eight items). The answers follow a five-point Likert scale ranging from 1 to 5 (from never to every day). There is the burnout syndrome in the manifestation of high emotional exhaustion, high depersonalization and low personal fulfillment^(^ 1 ^-^ 4 ^)^.

The MBI-HSS has shown good internal consistency in studies carried out in several countries such as Spain, Mexico, Chile, Portugal, Colombia and Brazil, ranging from 0.79 to 0.91 for the *emotional exhaustion* dimension (α=0.90 in the original version); between 0.69 and 0.87 for *personal fulfillment* (α=0.71 in the original version) and between 0.42 and 0.66 for the *depersonalization* dimension (α=0.79 in the original version). This lower score for the depersonalization dimension has, frequently, occurred in studies conducted outside the United States^(^ 1 ^-^ 4 ^,^ 9 ^-^ 10 ^)^.

Confirmatory structural analysis is widely used to assess the relationship between items and factors in an instrument and in the international scientific literature there are studies that indicate from one to six factors for MBI-HSS^(^ 4 ^,^ 11 ^-^ 13 ^)^. Most of them indicate the original composition, with three factors/dimensions, as the most suitable^(^ 11 ^,^ 13 ^)^.

In Brazil, in recent years, studies on the factorial validity of the MBI for students (MBI-ES)^(^ 14 ^-^ 16 ^)^, health, justice, security and education professionals (MBI-GS)^(^ 15 ^)^, nephrology nurses (MBI-HSS)^(^ 15 ^)^ and nursing assistants (MBI-HSS)^(^ 17 ^)^ have been developed.

Given such context, even though the instrument is safe and measures what it is really wanted to measure, it is necessary to demonstrate how it behaved in this study, with this specific sample, in order to provide relevant data for the literature, which indicate that the instrument is consistent, does not have distortion in the measurement and remains with its three-factor structure according to the original version.

Considering the variability of results in the studies already carried out with MBI-HSS and the relevance of confirmatory analyses for the validation of this important measurement instrument in different populations and contexts, this study aims to confirm the factorial validity of the Maslach Burnout Inventory – Human Services Survey version in a sample of health professionals from the emergency services.

## Method

The study population corresponded to 840 health professionals (physicians, nurses, and nursing technicians), who work in mobile, pre-hospital, and hospital emergency services in a city in the inland of the state of São Paulo, Brazil. The inclusion criterion was a minimum service time of one year. Thus, the sample was calculated taking into account ten subjects for each variable, with the final N equal to 282 participants^(^ 18 ^)^.

All the ethical aspects were respected and the research project was approved by the institution’s Ethics Committee, with opinion number No. 1,266,959 and CAAE No. 47147815.0000.5993 according to Resolution No. 466/2012, which deals with research with human beings in Brazil.

For data collection, the participants of the Maslach Burnout Inventory – Human Services Survey (MBI-HSS), self-completed a Likert type scale type with 5 points and a sociodemographic questionnaire with 20 questions, namely: gender; date of birth; scholarship; marital situation; religion; occupation; position; service time; number of employment contracts; work shift; weekly workload; with whom do you live?; do you have children?; do you practice physical activity?; sleep duration; do you have a health problem?; do you use a psychotropic drug?; do you use anti-inflammatory drugs?; have you been away from work in the last year?, and: do you smoke?

Collection took place during the work shift, in a place reserved in the service itself, from October 2015 to March 2016. The data were analyzed using the additional specific AMOS module from the Statistical Package for the Social Sciences (SPSS) statistical program, version 19.0.

The psychometric sensitivity of the MBI-HSS items was estimated by measures of central tendency (mean and median), variability (standard deviation) and distribution shape. The latter being tested by the Shapiro-Wilk test, a result of p<0.05 was obtained. The internal consistency of each MBI-HSS factor was estimated using the standardized *Cronbach*’s alpha coefficient (α)*,* with α≥0.70 being considered adequate*.* The adequacy of the sample was verified using the Kaiser-Meyer-Olkin (KMO) index.

To test the data fit to the original three-factor structure proposed for the MBI-HSS, a confirmatory factor analysis (CFA) was performed, using the maximum likelihood method.

As indexes for assessing the quality of fit of the model, the chi-square ratio for degrees of freedom (χ^2^/DoF), the comparative fit index (CFI), the goodness of fit index (GFI), the Tucker Lewis Index (TLI) and root mean square error of approximation (RMSEA) were considered. The adjustment of the models was considered adequate when χ^2^/DoF≤5.0, CFI and GFI≥0.90 and with RMSEA values <0.08^(^ 18 ^)^.

To check for the existence of a correlation between the errors, the modification indexes were used from the Lagrange Multipliers. The comparison between the models was performed using indexes based on the Information Theory (Akaike Information Criterion - AIC, Browne-Cudeck Criterion - BCC and Bayes Information Criterion - BIC), the best model being considered the one that displayed the lowest values in these indexes^(^ 18 ^)^.

## Results

The sample consisted of 282 health professionals with a mean age of 40 years old (SD±9.4), mostly characterized by women (79.1%), married or with partners (52.1%) and with children (66.3%). As for schooling, 61.3% have high school and 38.7%, higher education. Considering their professions, 16% were nurses, 73.4% nursing technicians and 10.6% physicians. The mean time of work in the emergency services was 10 years (SD±8.2).

As for the characteristics related to the health of the professionals, 55% do not practice regular physical activity, the mean hours of sleep *per* night was 6.2 h (SD±1.3), 41.1% would report any health problem, 13.8% used psychotropic drugs, 49.3% used psychostimulants, 58.2% used anti-inflammatories and 37.6% reported being away from work in the last year.

Table 1 details the descriptive results of the instrument, regarding the psychometric sensitivity of the MBI-HSS items. It was considered that the absolute values of kurtosis (Ku<7) and asymmetry (Sk<3) did not indicate severe deviations from the normal distribution of the answers and, consequently, from psychometric sensitivity.

**Table 1:** Summary and distribution measures by items of the Maslach Burnout Inventory - Human Services Survey (MBI-HSS) for health professionals in emergency services (n=282). Ribeirão Preto, SP, Brazil, 2015-2016

Item	Mean	Median	Standard deviation	Kurtosis	Asymmetry
1	2.92	3.00	0.88	0.20	-0.06
2	3.17	3.00	0.86	-0.02	-0.27
3	2.80	3.00	1.05	-0.45	0.30
4	3.92	4.00	0.79	1.08	-0.68
5	1.52	1.00	0.77	-0.24	1.11
6	2.63	3.00	1.09	-0.49	0.25
7	4.30	4.00	0.76	3.93	-1.46
8	2.67	3.00	1.00	-0.28	0.17
9	3.62	4.00	1.04	0.27	-0.67
10	2.42	2.00	1.17	-0.76	0.33
11	2.26	2.00	1.10	-0.36	0.56
12	3.27	3.00	0.96	-0.41	-0.11
13	2.17	2.00	1.00	-0.52	0.40
14	3.02	3.00	0.98	-0.13	0.10
15	1.69	1.00	1.07	2.82	1.82
16	2.36	2.00	0.87	0.02	0.24
17	3.85	4.00	0.81	-0.05	-0.38
18	3.54	4.00	0.92	-0.16	-0.28
19	3.93	4.00	0.85	0.15	-0.64
20	2.21	2.00	1.02	-0.50	0.44
21	3.89	4.00	0.82	-0.21	-0.36
22	2.30	2.00	1.15	-0.64	0.47

The internal consistency of the MBI-HSS, considering the complete instrument, was adequate for the emotional exhaustion (α=0.879) and personal fulfillment (α=0.692) factors, being below the adequate for the depersonalization factor (α=0.594).

During the MBI-HSS internal consistency analysis process, it was verified that some items exceeded the alpha of the dimension to which they belong: 16 (α=0.88); 9 (α=0.693); 15 (α=0.663) and 22 (α=0.600). After eliminating these items from the KMO index, the best result (KMO=0.891) was obtained.

Subsequently, the confirmatory factor analysis began and, in Table 2, the results for the three models proposed for the MBI-HSS can be seen.

**Table 2:** Quality indexes for adjustment of the Confirmatory Factor Analysis (CFA) and Indexes based on the information theory (AIC, BIC and BCC) for health professionals in emergency services (n=282). Ribeirão Preto, SP, Brazil, 2015-2016

Estimates	Model 1*	Model 2^†^	Model 3^‡^
χ^2^	448.83	312.31	242.73
χ^2^/DoF	2.16	1.87	1.84
CFI^§^	0.86	0.91	0.93
GFI^||^	0.86	0.89	0.90
TLI^¶^	0.85	0.89	0.91
RMSEA**	0.07	0.06	0.06
AIC^††^	539.83	398.31	320.73
BIC^‡‡^	707.01	551.25	459.44
BCC^§§^	549.03	405.93	326.93

* Model 1 = Three-factor orthogonal model;

† Model 2 = Oblique, three-factor model;

‡ Model 3 = Second-order hierarchic model;

§ CFI = Comparative Fit Index;

|| GFI = Goodness of fit index;

¶ TLI = Tucker Lewis index;

** RMSEA = Root mean square error of approximation;

†† AIC = Akaike information criterion;

‡‡ BIC = Baves information criterion;

§§ BCC = Browne-Cudeck criterion

Model 1, initial model following the three-factor proposal of the original MBI-HSS, adjusted for the sample of health professionals working in the area of urgency and emergency, revealed a quality of adjustment that can be considered unsatisfactory, according to the values obtained χ^2^/DoF = 2.16; CFI = 0.86; GFI = 0.86; TLI = 0.85 and RMSEA = 0.07.

In order to achieve a better fit of the model, the original model was refined according to the modification indexes obtained through AMOS. In this first phase, item 12 of the *personal fulfillment* dimension and item 16 of the *emotional exhaustion* dimension were deleted due to their modification indexes suggesting such a correction.

In Model 2, after deleting items 12 and 16, a more satisfactory fit quality was obtained, according to the values obtained in the indexes χ^2^/DoF = 1.87; CFI = 0.91; GFI = 0.89; TLI = 0.89 and RMSEA = 0.06. However, it was observed that item 9 of the *personal fulfillment* dimension and item 15 of the *depersonalization* dimension still had factorial weights below what was considered adequate (≥0.40) and needed to be removed from the model.

**Figure 1:**
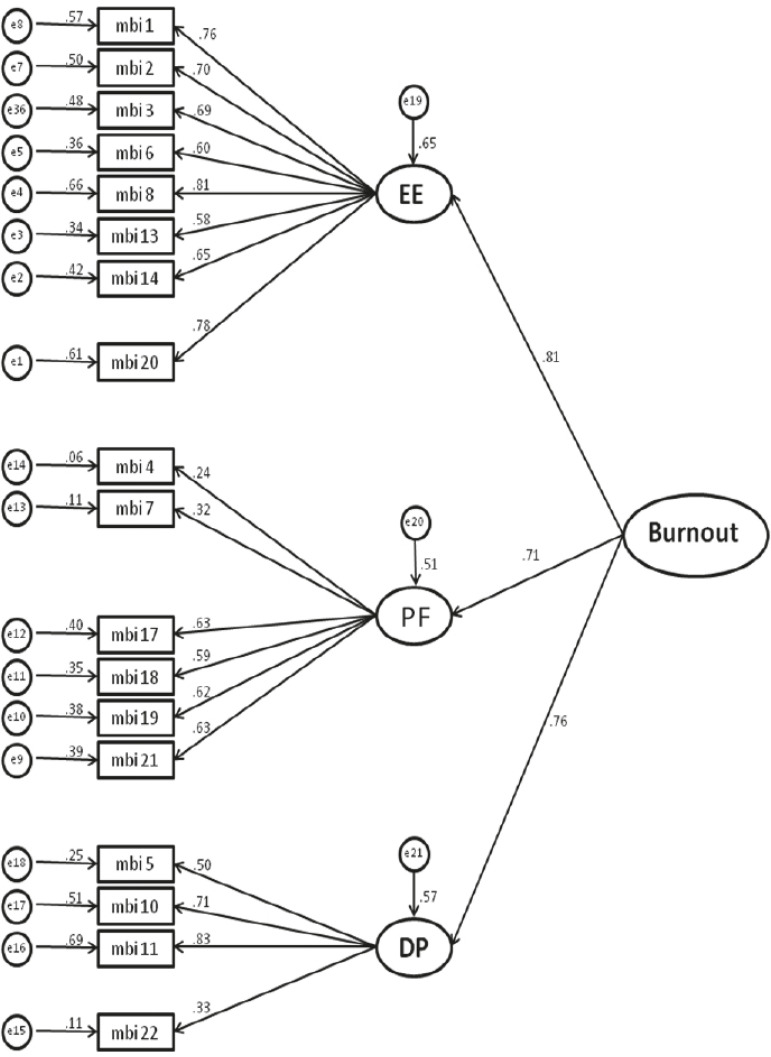
Second-order hierarchical model of the MBI-HSS adjusted for the sample of health professionals in emergency services (n=282). Ribeirão Preto, SP, Brazil, 2015-2016 EE = Emotional exhaustion; PF = Professional fulfillment; DP = Depersonalization

With the removal of items 9 and 15 Model 3 was obtained, with indexes χ^2^/DoF = 1.84; CFI = 0.93; GFI = 0.90; TLI = 0.91 and RMSEA = 0.06, considered the best in relation to the three models, which arrived at the second order hierarchical model with the mentioned modifications (Figure 1), presented adequate adjustment to the data and can be considered the best and most parsimonious model tested according to the information theory indexes (AIC = 320.73; BIC = 459.44; BCC = 326.93). The internal consistency of the three dimensions of the MBI-HSS was recalculated considering the exclusion of the items and were considered adequate according to the literature (*Emotional exhaustion*: α = 0.88; *Depersonalization*: α = 0.66; *Professional fulfillment*: α = 0.67). Model 4 is shown in Figure 1.

## Discussion

The results obtained in this sample confirm the theoretical model of the burnout syndrome with regard to the constitution of three dimensions as proposed by the original American inventory^(^ 1 ^)^, as well as in studies carried out in other countries, in which the factorial structure found was maintained as in the original version^(^ 4 ^,^ 18 ^-^ 21 ^)^.

The internal consistency of the MBI-HSS for health professionals in emergency services was considered adequate for the emotional exhaustion and personal fulfillment factors, but it was below the adequate for the depersonalization factor.

Similar results were found in studies developed in other countries^(^ 4 ^,^ 20 ^-^ 25 ^)^, indicating that, despite the cultural differences, the scale has maintained its cross-cultural validity^(^ 26 ^)^. Normally, indexes have been found between 0.71 and 0.91 for the emotional exhaustion dimension, between 0.69 and 0.87 for personal fulfillment and between 0.42 and 0.64 for depersonalization^(^ 3 ^,^ 20 ^-^ 25 ^)^.

The *emotional exhaustion* dimension stands out as the least vulnerable to cultural issues^(^ 26 ^)^. This result may indicate a more universal relationship between the statements that make up the emotional exhaustion dimension and the feeling of weariness due to work that occurs in individuals (relationship between the dimension and the construct).

The low internal consistency of the *depersonalization* dimension, in comparison with the other two dimensions of emotional exhaustion and personal fulfillment, is often found in other studies^(^ 3 ^-^ 4 ^,^ 11 ^,^ 25 ^)^. This result may be related to the small number of items that make up this dimension^(^ 11 ^)^. In addition, social demands can be a burden for the health professionals, whose main objective is to care for others. Admitting certain level of depersonalization can pose a psychological threat and interfere with their levels of self-esteem and perceived self-efficacy, as feelings of depersonalization refer to certain distancing from the service recipients.

Despite the lower internal consistency of the factors that make up the depersonalization dimension and the lack of personal fulfillment in the face of emotional exhaustion, the latter is considered, according to the scale’s own authors, the most important and the one that really reflects emotional strain^(^ 8 ^)^. Also according to the authors, depersonalization can act as a coping strategy in a situation of exhaustion, while the lack of personal fulfillment would be a consequence^(^ 3 ^)^. Therefore, maintaining it in the instrument is relevant and was adopted in this study.

It was possible to obtain an adequate factorial solution, with satisfactory adjustment levels, paying attention to the psychometric sensitivity of the items in the sample. All the items that make up the MBI-HSS showed adequate asymmetry and kurtosis, allowing asserting that, in general, they fit well to a normal distribution being able to, adequately, discriminate different levels of evaluation.

Considering the factorial loads of the items that make up the MBI-HSS scale, it is possible to verify that the same has occurred in other studies^(^ 21 ^,^ 26 ^)^ and such loads may be related to the sample characteristics, the scale design or the cultural factors.

Despite the satisfactory adjustment of the three models tested for the MBI-HSS for health professionals in emergency services, Model 3 was considered the most parsimonious and the model of choice for continuing the process of validating the factorial structure of the MBI-HSS in this sample. It was chosen to consider the correlations between errors detected by the modification indexes and four items had a factorial weight below what was considered adequate.

Thus, the following items were removed from the model: item 16 of the *emotional exhaustion* dimension; items 9 and 12 of the *personal fulfillment* dimension and item 15 of the *depersonalization* dimension, due to their factor-related weight below the one considered adequate.

There is no consensus regarding the items that should be excluded from the scale. However, some items are known to be more ambiguous, as is the case of items 12 and 16, as well as items 2, 6 and 20^(^ 4 ^,^ 11 ^,^ 19 ^,^ 21 ^)^. The scale’s authors themselves suggested not considering items 12 and 16 in confirmatory factorial studies^(^ 22 ^,^ 27 ^)^. In this sense, different studies have shown that, due to their inadequate factor weight, some items end up being removed.

A number of studies carried out in Latin America corroborate the results obtained. In Peru, a research carried out with nurses removed a total of seven items from the original scale, leaving the final version with 15 items^(^ 4 ^)^. In a Chilean study, conducted with professionals from different health services, items 5, 13, 20, and 21 were excluded because they had an inadequate factorial weight, as well as items 12 and 14 due to factorial ambiguity. Thus, the 17-item version replicates the theoretical model of the expected relationships between the factors^(^ 21 ^)^. Another investigation, conducted with Nicaraguan teachers of basic and high school education from 112 rural and urban educational centers, concluded that the corrected version with 13 items had higher rates than the original scale consisting of 22. Furthermore, this study concluded that 41% of the items in the scale did not correctly saturate the established factorial loads and showed an inadequate factorial weight, thus evidencing that the MBI construct, as planned, does not show enough weight to assume its universality among heterogeneous cultures^(^ 22 ^)^.

In an Argentinian study conducted with mental health professionals, it was observed that the adjustment of the model improved satisfactorily, confirming the three-factor structure after removing item 12. This item obtained a satisfactory factorial load in the three dimensions, behaving in an ambiguous way; the same was verified in the original study^(^ 19 ^)^. In Córdoba, a study conducted with professionals from different work services tested six models that varied from one to four factors. The best adjustment occurred with Model 2, with the removal of three items: 3, 8 and 13^(^ 21 ^)^.

A multi-center study carried out with 2,470 health care professionals from Bolivia, Colombia, Costa Rica, Ecuador, Mexico, Peru, Dominican Republic and Venezuela, excluded item 8 for better adjustment of the three-factor model, due to its low factorial weight^(^ 25 ^)^.

In Brazil, a research conducted with bank employees showed a four-factor structural model composed of 19 items. Eight models were tested and, after exclusion of items 4, 7 and 22 for presenting inappropriate factorial behavior to the matrix, model eight presented the best fit^(^ 28 ^)^. Another Brazilian study with a sample of nursing assistants, divided between groups with major depressive disorder and absence of the disorder, found that the acceptable rates for RMSEA and CFI occurred in the group with no disorder. And in the general sample of the study, the three-factor model proved to be acceptable, but concluding that the two-factor model was better adjusted in this sample^(^ 29 ^)^.

In the same sense, a number of European studies showed the relevance of confirmatory analysis studies for the scale in different samples. A Hungarian study conducted with elementary and high school teachers tested eight models, with the best fit obtained with the bi-factorial model^(^ 30 ^)^. A Spanish investigation, conducted with a sample of social workers, supported the superior three-factor model compared to alternative models of one or two factors. In addition, items 12, 13 and 16 were excluded, which favored better adjustment and internal consistency to MBI-HSS in the referred sample^(^ 31 ^)^.

Another study, conducted with Australian lawyers, concluded that the five-factor structure better explains the multi-factorial nature of the burnout syndrome^(^ 18 ^)^. In a study conducted in Thailand with Medicine post-graduate students, identified that the three-factor model obtained values considered acceptable, after data adjustment^(^ 23 ^)^.

In turn, with respect to items 9 and 15 excluded in this study and not associated with any of the three dimensions of the scale, no studies with similar results were identified in the scientific literature. Item 9 refers to the perception of the professional to be positively influencing other people’s lives through their work. As for item 15, it can be that the health professionals have had difficulty in admitting, really, not caring about “what happens to some” patients, which can justify the result obtained.

These factorial differences of some MBI items in different samples and cultural contexts show the relevance of validation studies in different populations, demonstrating the need for rigor in the adaptation of the instrument, considering its content, language adopted, cultural reality, context, and homogeneity of the samples investigated.

Despite the removal of four items from the original scale, the final instrument that resulted from the analysis of this study, consisting of 18 items, replicates the theoretical model of the authors of the scale and reproduces the expected theoretical relationships between the factors. The internal consistency of the three factors of the MBI-HSS for health professionals in emergency services was recalculated considering the exclusion of the items and was considered adequate. If compared to the original version, the final version obtained has 18 items now, with better internal consistency, considering the Brazilian reality of the health professionals working in the emergency services.

As limitations, although the instrument has some weaknesses in the *depersonalization* dimension, it has a satisfactory internal consistency, particularly in the *emotional exhaustion* and *personal fulfillment* dimensions, which confirms its psychometric quality.

The behavior of a scale in one sample does not guarantee the same behavior in other samples, so it is desirable that they be confirmed in other studies, exploring new samples in order to arrive at a more consistent conclusion about a psychometric instrument.

The present study confirms the factorial validity of MBI-HSS for health professionals in emergency services and corroborates with other researchs that prove it as one of the most used instruments in empirical research studies on workers’ health, with greater international diffusion, reliable and relevant for burnout syndrome assessment. 

## Conclusion

The confirmatory factor analysis of the MBI-HSS instrument for measuring burnout syndrome in health professionals from the emergency services signals that the Brazilian version of the instrument follows a three-factor structure, as in the original version, with adequate internal consistency of the items. This outcome contributes to the scientific validation of the instrument and provides greater safety for its use by researchers on this them and similar samples.

The Brazilian version of the MBI-HSS for health professionals from the emergency services meets all the necessary requirements in terms of internal consistency and structural validity to be widely used in the assessment of the burnout syndrome in this context. Researchers in the field are recommended to consider studies already carried out and the existing updated evidence for planning and carrying out new studies with the MBI-HSS.
